# Retrospective Genomic Surveillance of Chikungunya Transmission in Minas Gerais State, Southeast Brazil

**DOI:** 10.1128/spectrum.01285-22

**Published:** 2022-08-25

**Authors:** Hegger Fritsch, Marta Giovanetti, Joilson Xavier, Talita Emile Ribeiro Adelino, Vagner Fonseca, Jaqueline Góes de Jesus, Ronaldo de Jesus, Carla Freitas, Cassio Roberto Leonel Peterka, Carlos Frederico Campelo de Albuquerque, Ana Maria Bispo de Filippis, Rivaldo Venâncio da Cunha, Erniria Carvalhais Silva, Luiz Carlos Junior Alcantara, Felipe Campos de Melo Iani

**Affiliations:** a Laboratorio de Flavivírus, Instituto Oswaldo Cruz, Fundação Oswaldo Cruz, Rio de Janeiro, Brazil; b Department of Science and Technology for Humans and the Environment, Campus Bio-Medico University of Rome, Rome, Italy; c Laboratório Central de Saúde Pública do Estado de Minas Gerais, Fundação Ezequiel Dias, Belo Horizonte, Minas Gerais, Brazil; d Organização Pan-Americana da Saúde, Organização Mundial da Saúde, Brasília, Distrito Federal, Brazil; e Laboratório de Patologia Experimental, Instituto Gonçalo Moniz, Fundacão Oswaldo Cruz, Salvador, Brazil; f Coordenação Geral dos Laboratórios de Saúde Pública, Secretaria de Vigilância em Saúde, Ministério da Saúde, Brasília, Distrito Federal, Brazil; g Coordenação Geral das Arboviroses, Secretaria de Vigilância em Saúde, Ministério da Saúde (CGARB/SVS-MS), Brasília, Distrito Federal, Brazil; h Fundação Oswaldo Cruz, Bio-Manguinhos, Rio de Janeiro, Brazil; i Coordenadoria Estadual de Vigilância das Arboviroses, Secretaria de Estado de Saúde de Minas Gerais, Belo Horizonte, Minas Gerais, Brazil; Emory University School of Medicine

**Keywords:** CHIKV, genomic monitoring, southeast Brazil, Nanopore sequencing

## Abstract

Brazil accounted for a total number of 1,276,194 reported cases of chikungunya fever between 2014 and 2022. Additionally, since 2015, the country has experienced an increasing death toll, in which the Northeast and Southeast regions appear to report the worst scenarios. Although the CHIKV transmission dynamics have been studied in many parts of the country since its introduction in 2014, little is still known about chikungunya virus (CHIKV) transmission and genetic diversity in the state of Minas Gerais, located in southeast Brazil. Moreover, no studies have been published characterizing CHIKV genomic surveillance in this state. Thus, to retrospectively explore the CHIKV epidemic in Minas Gerais, we generated 40 genomes from clinical samples using Nanopore sequencing. Phylogenetic analysis indicated that multiple introductions of CHIKV occurred, likely from the northeastern Brazilian states, with the most recent common ancestral strain dating to early March 2016, which is in agreement with local epidemiological reports. Additionally, epidemiological data reveals a decline in the number of reported cases from 2017 to 2021, indicating that population immunity or changes in vector activity may have contributed to the decreasing waves of CHIKV infection. Together, our results shed light on the dispersion dynamics of CHIKV and show that infections decreased from March 2017 to January 2021 despite multiple introductions into Minas Gerais State. In conclusion, our study highlights the importance of combining genomic and epidemiological data in order to assist public health laboratories in monitoring and understanding the patterns and diversity of mosquito-borne viral epidemics.

**IMPORTANCE** Arbovirus infections in Brazil, including chikungunya, dengue, yellow fever, and Zika, result in considerable morbidity and mortality and are pressing public health concerns. However, our understanding of these outbreaks is hampered by the limited availability of genomic data. In this study, we combine epidemiological analysis and portable genome sequencing to retrospectively describe the CHIKV epidemic in Minas Gerais between 2017 and 2021. Our results indicate that the East/Central/South African (ECSA) CHIKV lineage was introduced into Minas Gerais by three distinct events, likely from the North and Northeast regions of Brazil. Our study provides an understanding of how CHIKV initiates transmission in the region and illustrates that genomics in the field can augment traditional approaches to infectious disease surveillance and control.

## INTRODUCTION

Chikungunya virus (CHIKV) is an alphavirus belonging to the family *Togaviridae* that is transmitted through the bite of infected mosquitoes of the genus *Aedes* (1–4). CHIKV can be classified in four distinct lineages (or genotypes): (i) the West African lineage; (ii) the East/Central/South African (ECSA) lineage; (iii) the Asian lineage, and (iv) the Indian Ocean lineage (IOL) (5–7). CHIKV infection is characterized by high fever (>38.9°C), followed by the occurrence of cutaneous manifestations, fatigue, myalgia, and a debilitating polyarthralgia. Patients may also develop chronic and potentially incapacitating rheumatic musculoskeletal disorders known as chronic chikungunya arthritis, which currently represent an important public health burden (2, 8, 9).

An alarming number of infections have been reported worldwide since the first case in Tanzania in 1953 (6, 10, 11). From 2013 to 2022, more than 3,202,355 probable cases and 276,852 laboratory-confirmed cases were reported in the Americas (12), of which Brazil comprised the highest incidence rates and number of cases in the continent (12). In Brazil, local transmission of the CHIKV East/Central/South (ECSA) genotype was detected for the first time in the municipality of Feira de Santana in 2014, nearly simultaneously with the introduction of the Asian lineage into Oiapoque, Amapá State, in the northern region (13). Since then, the ECSA genotype has been detected in several other Brazilian states, located in the northeastern, southeastern, northern, and midwestern regions, and beyond Brazil’s borders (Paraguay and Haiti), representing a serious threat to public health (13–16), especially because CHIKV-ECSA infections are associated with higher symptomatic manifestations compared to other genotypes (17).

CHIKV infections in Brazil accounted for 1,276,194 suspected cases between 2014 and 2022, most of which were reported in the northeastern and southeastern regions (12, 18–22). In 2016, the number of CHIKV cases in the southeast region reached a total of 18,691, driven by Rio de Janeiro State, which accounted for 75.1% of cases from that region. In that same period, the state of Minas Gerais reported 1,292 cases, which corresponds to 6.9% of all cases from the southeast region. However, the epidemic transmission and genetic diversity of CHIKV in Minas Gerais remain poorly understood, as the paucity of complete genomic sequences available impairs our understanding of the introduction and establishment of CHIKV in that state. Thus, in this study, we used Nanopore sequencing to generate 40 genomes, sampled between 2017 and 2021 from infected patients residing in Minas Gerais, and provide a retrospective reconstruction of the transmission dynamics in that state.

## RESULTS

**FIG 1:**
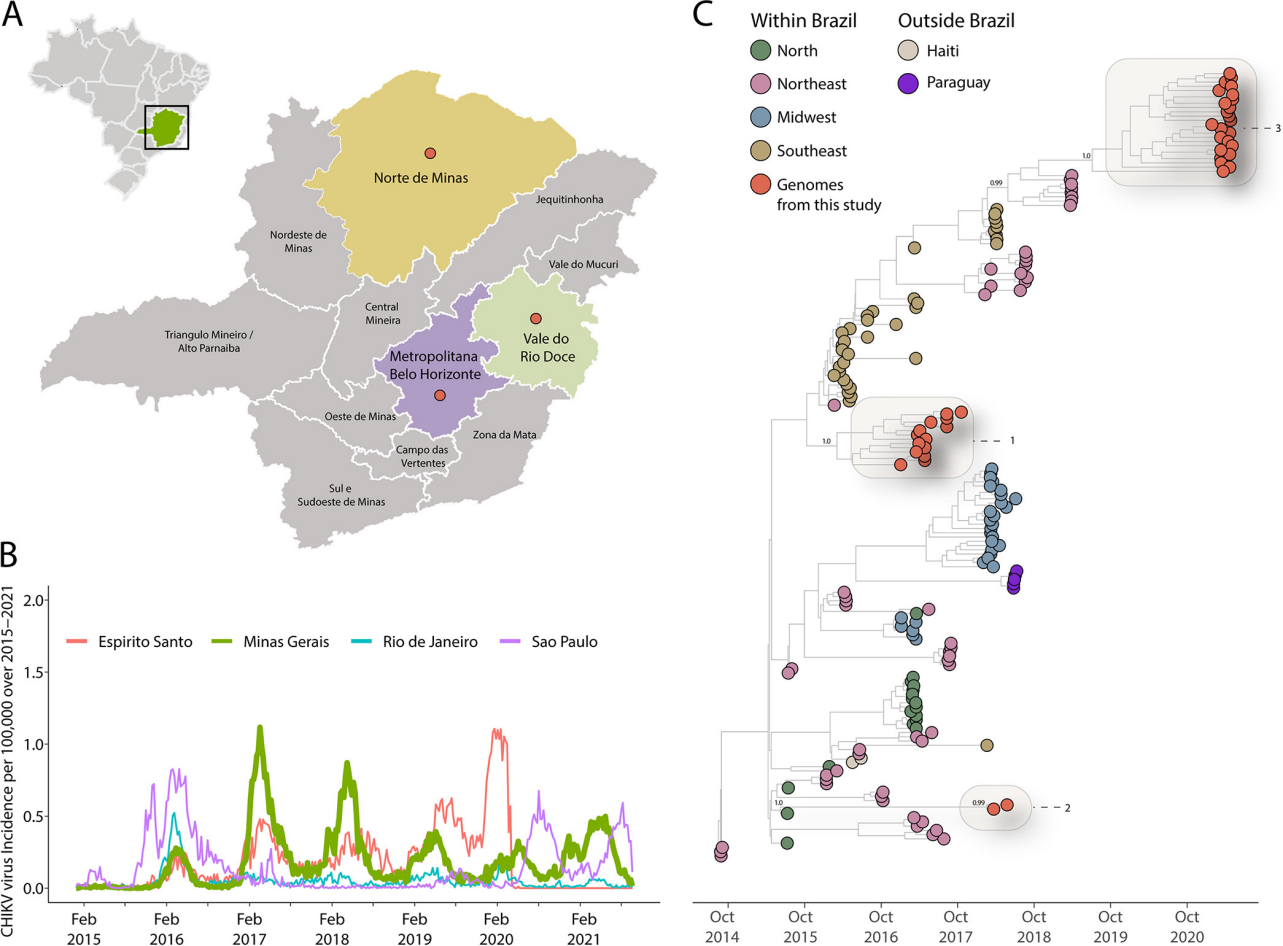
Genomic monitoring of CHIKV in Minas Gerais, southeast Brazil. (A) Map of Brazil and Minas Gerais showing the spatial area under investigation. (B) Weekly reported Chikungunya cases normalized per incidence per 100,000 inhabitants. The *y* axis values were log-transformed for visualization purposes. (C) Time-scaled maximum clade credibility phylogeny of the CHIKV-ECSA lineage, including the 40 new genomes generated in this study plus 145 reference strains. Tips are colored according to the sample source location. Values around the key nodes represent the posterior probability support.

To retrospectively investigate the CHIKV-ECSA epidemic in Minas Gerais, Brazil, we generated 40 genomes using Nanopore sequencing, collected from 53% female and 47% male patients with a median age of 40 years (range, 3 months to 83 years) (Table 1). All sequenced samples were collected from different municipalities in the state of Minas Gerais (Fig. 1A; see Table S1 in the supplemental material) and contained sufficient viral genetic material (≥2 ng/μL) for library preparation. The reverse transcriptase quantitative PCR (RT-qPCR) threshold cycle (*C_T_*) values were on average 17.88 (range, 5.37 to 29.92), and the sequences presented a median genome coverage of 83.90% (range, 59.9% to 93.2%). The epidemiological data and sequencing statistics are detailed in Table 1 (see also Table S1). The most reported symptoms were myalgia (90%), fever (85%), and arthralgia (80%), followed by back pain (60%) and headache (55%). We also identified 8 cases of retro-orbital pain (40%) and 4 (20%) of petechiae at lower frequencies (Table 1). For those who mentioned previous comorbidities, chronic kidney injuries were reported by 4 patients (20%) (Table 1).

**TABLE 1:** Demographics and clinical symptoms of patients with Chikungunya fever

Characteristic	Data
Demographics (*n* = 40)	
Mean age (yrs)	41.60 ± 22.53
Gender	
Female	21 (52.5%)
Male	19 (47.5%)
Mean no. of days with symptoms before diagnosis (SD)	2.72 ± 2.68
CHIKV RT-qPCR mean *C_T_* value	17.88 ± 5.92
Clinical data (*n* = 20)	
Presenting symptoms	
Fever	17 (85%)
Myalgia	18 (90%)
Headache	11 (55%)
Cutaneous rash	2 (10%)
Vomiting	2 (10%)
Nausea	3 (15%)
Back pain	12 (60%)
Arthritis	1 (5%)
Arthralgia	16 (80%)
Petechiae	4 (20%)
Retro-orbital pain	8 (40%)
Comorbidities	
Diabetes	1 (5%)
Liver injury	1 (5%)
Chronic kidney injury	4 (20%)

Figure 1B shows the CHIKV weekly cases normalized per 100,000 individuals reported between 2015 and 2021 in the southeastern region of Brazil (Minas Gerais, Rio de Janeiro, Espirito Santo, and Sao Paulo states). After its first introduction, the virus spread unnoticed until the first increase in incidence in February 2016. In 2017 and 2018, the state of Minas Gerais faced two major outbreaks, possibly related to the importation from the northeastern region, as indicated by the increase in incidence reported in northeastern states around the same period and also by the close phylogenetic relationship between the sequences (Fig. 1C; Fig. S1). These prominent peaks might be associated with an elevated number of pathogen-naive and susceptible individuals. After mid-2018, the incidence of new cases was smaller than in the previous outbreaks, which might be the result of an increase in the number of people with a protective immunological response due to previous exposure or infection in that state. While Minas Gerais exhibited a reduction in case numbers, other southeastern states, such as Espirito Santo, reported an increase in CHIKV incidence in 2019 and 2020, as indicated by a peak in the epidemic curve in Fig. 1B. Despite a reduction in the incidence, we can observe a resurgence in new CHIKV cases reported in Minas Gerais between 2019 and 2021, as indicated by the epidemic curves (Fig. 1B).

To explore the relationship of the CHIKV genomes generated in this study to those of other isolates, a combined data set was subjected to phylogenetic inference. A regression in the genetic divergence from root to tip against the sampling dates confirmed a sufficient temporal signal (correlation coefficient = 0.85; *R*^2^ = 0.72). Our maximum clade credibility (MCC) tree suggested that at least three independent introduction events occurred in the state of Minas Gerais, as indicated by three clades in Fig. 1C that also clustered with viruses isolated in other Brazilian regions (Northeast and North), suggesting that those regions likely acted as a stepping-stone for the dissemination of the virus into the state of Minas Gerais (Fig. 1C). From our time-measured tree, we estimated that the most recent common ancestor (TMRCA) of the CHIKV epidemic in Minas Gerais occurred around early March 2016 (95% highest posterior density [HPD], early September 2015 to early June 2016) for the first estimated introduction event and in early October 2017 (95% HPD, early April 2017 to mid-February 2018) for the second event. Our results further revealed that the most recent CHIKV outbreak in Minas Gerais (clade 3) is likely related to a possible importation mediated by the Northeast Region (Rio Grande do Norte State) that may have occurred in early July 2019 (95% HPD, early October 2018 to early November 2019) (Fig. 1C).

## DISCUSSION

Genomic surveillance of arboviruses, including dengue virus, in clinical samples, as discussed in this work, was possible thanks to previous training activities in portable field sequencing and data analysis carried out with the team from the LACEN Minas Gerais. These activities, which allowed us to improve our understanding of molecular epidemiology and the dispersion of arboviruses circulating in Brazil, were promoted with the support of the Ministry of Health of Brazil and PAHO (23).

In this study, using portable Nanopore sequencing, we generated 40 new CHIKV genome sequences from samples collected between 2017 and 2021 in Minas Gerais State. The genomic data obtained in this work allowed us to retrospectively reconstruct and characterize the introductions of CHIKV in the state of Minas Gerais. Our data suggested that the circulation of the CHIKV-ECSA lineage in Minas Gerais may have resulted from at least three independent introduction events. The first event likely occurred around late 2016, followed by a second importation event (MG-02 clade) around early October 2017 closely related to sequence from the North Region, although data are scarce. Even though our early CHIKV genome from Minas Gerais was sampled in January 2017, there were reports of imported CHIKV cases in previous years (24, 25). The first report of autochthonous CHIKV cases in the state date to mid-March 2016 (25), when a clear increase in the incidence was also reported, resulting in the epidemic curve in 2016. These reports corroborate our estimate of the first CHIKV-ECSA clade dating to early March 2016, as observed in our time-scaled phylogeny.

By analyzing the number of reported CHIKV cases per year since the introduction of the virus into Brazil, we can observe a clear downward trend in the case numbers in most regions, mainly in the North and Northeast regions from 2018 to 2020. In 2019, when a new resurgence in CHIKV cases was reported in the Northeast and Southeast regions, there was again an increase in case numbers in Mina Gerais, which might represent the introduction of the common ancestor of clade 03, whose transmission persisted until 2021. Time-measured phylogenetic analysis suggested that clade 03 originated from an importation event from the Northeast Region after a series of virus exchanges between northeastern and southeastern states, with a pattern already observed for the ECSA lineage (14).

Since its introduction into Brazil, the high risk of dissemination of the ECSA lineage across the country has already been estimated, with the probable participation of human mobility as a driver of dispersion to other locations (13). In addition to human circulation, the distribution of competent mosquito vectors and climatic conditions may be closely associated with the extensive distribution of the CHIKV-ECSA lineage in the country, shortly after its introduction in the Northeast Region (13, 26). Accordingly to available data and epidemiological records, it seems that the Northeast and Southeast regions may stand out as the possible main sources of viral dissemination to other locations and nearby states. However, additional phylogeographic analyses should be performed to determine the viral spread between regions. The introduction of new viruses or variants, mediated by human mobility, as shown in this study, likely changes the landscape of local viral genetic diversity, with implications for epidemiology and public health (27).

### Conclusion.

Together, our results indicate that the CHIKV-ECSA lineage was introduced into Minas Gerais by three distinct events, likely from the North and Northeast regions of Brazil. These data shed light on the epidemiological dynamics of the virus circulating between regions and their impact on the origin of new outbreaks. In conclusion, our study highlights the importance of combining genomic data with accurate epidemiological and environmental information in order to assist public health laboratories in monitoring and understanding the patterns and diversity of mosquito-borne epidemics.

## MATERIALS AND METHODS

### Ethics statement.

This research was reviewed and approved by the Ethical Committee of the Pan American World Health Organization (number PAHO-2016-08-0029), the Oswaldo Cruz Foundation Ethics Committee (CAAE, 90249218.6.1001.5248), and the Brazilian Ministry of Health (MoH) as part of arbovirus genomic surveillance efforts within the terms of Resolution 510/2016 of CONEP (Comissão Nacional de Ética em Pesquisa, Ministério da Saúde [National Ethical Committee for Research, Ministry of Health]). Residual anonymized clinical diagnostic samples, with no or minimal risk to patients, were provided for research and surveillance purposes within the terms of Resolution 510/2016 of CONEP.

### Diagnostic procedures.

Serum from all patients with CHIKV symptoms treated by public health services in Minas Gerais State were collected from 2017 to 2021 for molecular diagnostics at the Central Laboratory of Public Health of Minas Gerais State (LACEN-MG) in southeast Brazil. After the molecular screening, a total of 40 positive serum samples with available epidemiological metadata, such as the date of symptom onset, date of sample collection, sex, age, and municipality of residence, were selected for whole-genome sequencing. The serum samples were submitted to nucleic acid purification using the MagMAX pathogen RNA/DNA kit and the KingFisher Flex purification system (Thermo Fisher), following the manufacturer’s recommendations. Detection of CHIKV RNA by RT-qPCR was performed using a protocol adapted from references 12 and 18–22, 28. Negative controls were used in all reactions.

### Synthesis of cDNA and whole-genome multiplex PCR.

Samples were selected for sequencing based on a *C_T_* value of <32 to maximize the genome coverage of the clinical samples by Nanopore sequencing (29). Positive samples were submitted to a cDNA synthesis protocol using the ProtoScript II First Strand cDNA synthesis kit (New England Biolabs). Then, multiplex PCR was conducted using Q5 Hot Start high-fidelity DNA polymerase (New England Biolabs) and a CHIKV whole-genome sequencing primer scheme (the primers are divided into two separate pools, A and B) (30). Thermocycling conditions previously described in reference 30 were used for 45 amplification cycles.

### Library preparation and sequencing procedures.

Whole-genome multiplex PCR was performed for both sequencing primer pools (A and B) separately (separated tubes). After PCR, the amplified products were purified using 1× AMPure XP beads (Beckman Coulter), and their concentrations were quantified using the Qubit double-stranded DNA (dsDNA) high-sensitivity (HS) assay kit on a Qubit 3.0 fluorimeter (Thermo Fisher). Then, the concentrations for each pool were normalized based on the previous quantification in a single tube. DNA library preparation was conducted for all samples which presented a DNA concentration of >1 ng/μL after the cleanup procedure. For the library preparation, the ligation sequencing kit (Oxford Nanopore Technologies) and native barcoding expansion kits 1-12 and 13-24 (Oxford Nanopore Technologies) were used, following the reaction conditions previously described in reference 30. One barcode was used for each sample, in order to optimize and increase the number of samples per flow cell in the same sequencing run. A sequencing library was generated using the SQK-LSK109 ligation sequencing kit (Oxford Nanopore Technologies) and loaded onto a R9.4 flow cell (Oxford Nanopore Technologies). Sequencing was performed for 8 h on a MinION device, and the final consensus sequences were obtained using Genome Detective software (https://www.genomedetective.com/) (31).

### Phylogenetic and Bayesian reconstructions.

The 40 newly sequenced genomes reported in this study (see Table S1 in the supplemental material) were initially submitted to a genotyping analysis using the phylogenetic arbovirus subtyping tool available at http://genomedetective.com/app/typingtool/chikungunya. Subsequently, the sequences were aligned with 145 complete or nearly complete CHIKV-ECSA genome sequences retrieved from NCBI through December 2021 (Table S2). Alignment was performed using MAFFT (32), and the sequences were manually curated to remove artifacts using AliView (32, 33). A maximum likelihood (ML) phylogenetic tree was estimated using IQ-TREE (34) under the Hasegawa-Kishino-Yano (HKY) nucleotide substitution model with 4 gamma categories (HKY+G4), which was inferred in jModelTest2 (https://github.com/ddarriba/jmodeltest2) (35). The robustness of the tree topology was determined using 1,000 bootstrap replicates, and the presence of temporal signals was evaluated using TempEst (36) through a regression of the root-to-tip genetic distances against sampling time. Time-scaled phylogenetic trees were inferred using the BEAST 1.10.4 package (37). We employed a stringent model selection analysis using both path-sampling (PS) and stepping-stone (SS) procedures to estimate the most appropriate molecular clock model for the Bayesian phylogenetic analysis (36–38). The uncorrelated relaxed molecular clock model was chosen as indicated by estimating the marginal likelihoods, also employing the codon-based SRD06 model of nucleotide substitution and the nonparametric coalescent Bayesian skyline model. We computed MCMC (Markov chain Monte Carlo) duplicated runs of 100 million states each, sampling 10,000 steps. Convergence of the MCMC chains was checked using Tracer 1.7.2 (38). The maximum clade (MC) tree was summarized using TreeAnnotator (http://beast.community/index.html), discarding 10% as burn-in.

### Epidemiological data assembly.

Data on the weekly reported CHIKV cases in Brazil between 2015 and 2021 were supplied by the Brazilian Ministry of Health and were plotted using R software 4.1.2 (http://www.r-project.org).

### Data availability.

The newly generated CHIKV sequences have been deposited at GenBank under accession numbers ON023487 to ON023526.
