# Supplementary material for: Retrospective Genomic Surveillance of Chikungunya Transmission in Minas Gerais State, Southeast Brazil
Source: Microbiol Spectr. 2022 Aug 25;10(5):e01285-22. doi: 10.1128/spectrum.01285-22 (PMC9602355; doi:10.1128/spectrum.01285-22)

S1 Fig. Chikungunya East-Central-South African lineage in Brazil total number of cases between the years of 2017 to 2021.

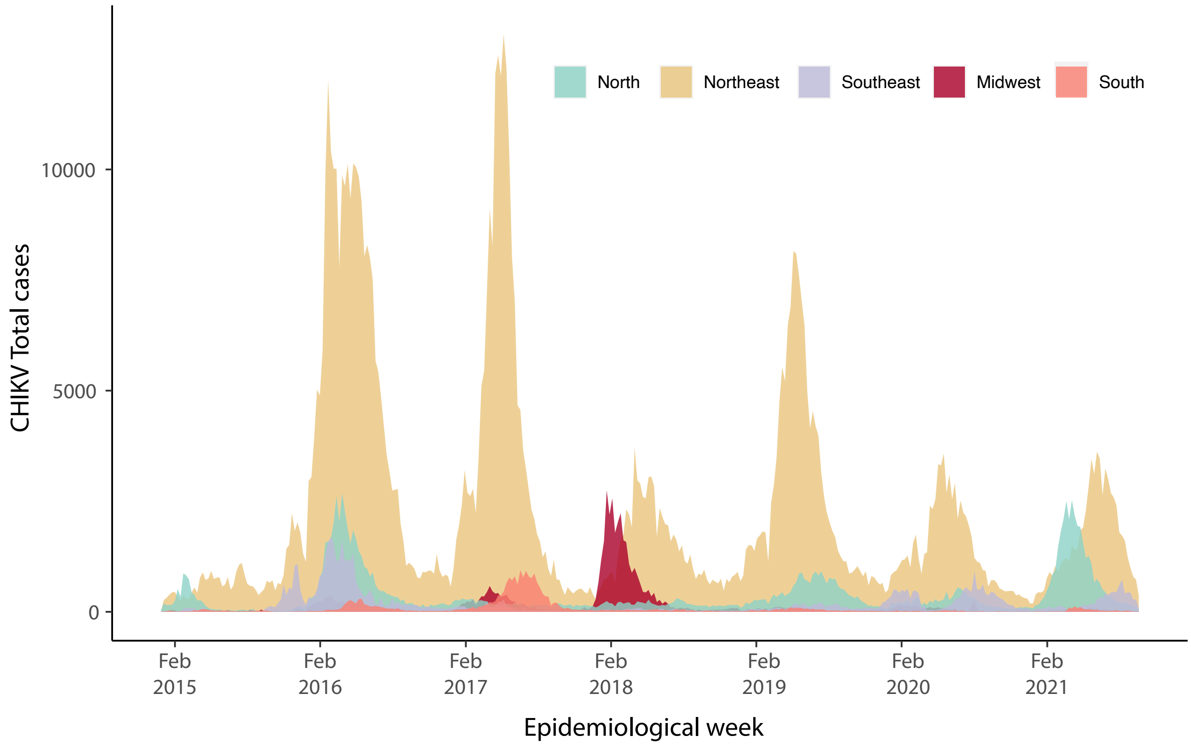

Supplement: Supplemental file 3 — Supplemental material. Download spectrum.01285-22-s0003.pdf, PDF file, 0.08 MB [file spectrum.01285-22-s0003.pdf]
